# Theoretical Methods for Studying DNA Structural Transitions under Applied Mechanical Constraints

**DOI:** 10.3390/polym9020074

**Published:** 2017-02-21

**Authors:** Artem K. Efremov, Ricksen S. Winardhi, Jie Yan

**Affiliations:** 1 Mechanobiology Institute, National University of Singapore, Singapore 117411, Singapore; 2 Centre for Bioimaging Sciences, National University of Singapore, Singapore 117557, Singapore; 3 Department of Physics, National University of Singapore, Singapore 117551, Singapore

**Keywords:** DNA, phase transition, equilibrium thermodynamics, force-spectroscopy, 87.14.gk, 87.15.La, 87.15.A-, 82.37.Rs, 05.70.Fh

## Abstract

Recent progress in single-molecule manipulation technologies has made it possible to exert force and torque on individual DNA biopolymers to probe their mechanical stability and interaction with various DNA-binding proteins. It was revealed in these experiments that the DNA structure and formation of nucleoprotein complexes by DNA-architectural proteins can be strongly modulated by an intricate interplay between the entropic elasticity of DNA and its global topology, which is closely related to the mechanical constraints applied to the DNA. Detailed understanding of the physical processes underlying the DNA behavior observed in single-molecule experiments requires the development of a general theoretical framework, which turned out to be a rather challenging task. Here, we review recent advances in theoretical methods that can be used to interpret single-molecule manipulation experiments on DNA.

## 1. Introduction

Deoxyribonucleic acid (DNA) is a fundamental biomolecule playing the major role in the preservation of genetic information across subsequent generations of living organisms. In nature, DNA can exist in several alternative forms, with the most important one being the DNA duplex consisting of two single-stranded polymers that are wound around each other forming a double helix structure. Both of the helix strands are made of a chain of basic building blocks, each containing a phosphate group, a deoxyribose sugar and one of four distinct nitrogenous bases: adenine (A), thymine (T), guanine (G) and cytosine (C). The architecture of the DNA duplex in general depends on the relative orientations of the bases, determining the way the two DNA strands interact with each other. At physiological conditions, DNA preferentially stays in the B-form, which is a right-handed duplex with a helical repeat of $h_{B}=10.4$ base-pairs (bp) and a base-pair rise of $\rho_{B}=0.33$ nm [1,2]. Highly specific formation of hydrogen bonds between A:T and G:C base-pairs of the opposite DNA strands, as well as the stacking interaction between aromatic rings of adjacent bases are the two major forces responsible for the stability of B-DNA.

Besides B-form, the DNA duplex can exist in several other states, including left-handed Z- and L-DNA with helical repeats of $h_{Z}∼12$ bp [3] and $h_{L}∼16$ bp [4,5,6], elongated right-handed underwound S-DNA ($h_{S}∼35$ bp [4,7,8,9]) and overwound P-DNA ($h_{P}∼3$ bp [4,9,10,11,12]). Each of these DNA structures has its own architecture, leading to very distinct mechanical properties. While the detailed molecular mechanisms causing DNA transition from one form to another are not completely understood, the existing experimental data suggest that the relative stability of these structures depends on several biochemical factors, including DNA nucleotide sequence [5,6,13,14,15,16,17] and surrounding environmental conditions, such as solution ionic strength [13,17,18,19] and the level of DNA hydration [20]. More importantly, numerous single-DNA manipulation experiments suggest that the DNA transition between alternative structural states can be strongly modulated by mechanical constraints applied to the DNA duplex [4,5,6,7,8,9,10,11,16,18,19,21,22,23,24,25,26,27].

Indeed, it has been shown in in vitro experiments that if torque exerted on DNA surpasses a certain threshold value, the DNA frequently undergoes conformational changes to reduce the accumulated twist elastic energy either by collapsing into a supercoiled configuration or switching into an alternative structural form [4,5,6,9,10,11,21,22,23,28]. In the former case, DNA typically develops plectonemes, structures in which DNA is twisted into a helical braid-like conformation [29,30]; whereas, in the latter scenario, DNA experiences transition from B- to L- and P-DNA forms. While L-DNA is a left-handed duplex that forms when DNA is placed under sufficiently large negative torsional stress (≲−10 pN·nm) [4,5,6], at high positive torques (≳35–40 pN·nm), DNA switches into a right-handed P-DNA state [4,11]. Furthermore, single-DNA manipulation studies show that if the force applied to a torsionally-relaxed DNA exceeds ∼65 pN, DNA undergoes overstretching transition from B- to S-DNA state [7,8]. Therefore, DNA conformation can be subject to various mechanical constraints, which may take place in living cells.

Existing experimental studies demonstrated that DNA packaging into a highly ordered chromatin structure inside living cells, as well as translation of the genetic information coded by DNA, are performed by numerous DNA-binding proteins, which frequently distort the DNA structure by inducing mechanical deformations of the DNA duplex [31,32,33,34,35,36,37,38,39,40,41,42,43]. Furthermore, an increasing body of evidence has shown that not only cells impose mechanical constraints on the chromosomal DNA at normal physiological conditions to control its global conformation, but more importantly, it has been revealed that living cells can actually sense and process various forces and torques exerted on the DNA to make critical decisions. For example, several recent experimental studies have demonstrated that mechanical forces applied to the nucleus may result in transcriptional activation of specific genes [44,45,46,47]. However, the molecular mechanisms by which living cells sense the applied force and respond to it by regulating the respective intracellular processes remain unclear.

To better comprehend the physiological functions of various mechanical constraints exerted on the chromosomal DNA that may exist in living cells, it is necessary to understand how the structure of DNA and its interaction with DNA-binding proteins are regulated by different mechanical stimuli. Current biochemical bulk methods are not suited to address such questions since they do not allow the application of mechanical constraints to DNA. On the other hand, recently-developed single-DNA manipulation techniques have made it possible to directly assess the DNA interaction with various proteins under different force and torque constraints. However, the molecular mechanisms responsible for the modulation of protein binding to DNA in response to these constraints are still not fully understood. To obtain more detailed insights into the underlying physical processes, the development of a general theoretical framework describing the DNA structural stability and DNA-protein interaction is required to interpret the experimental results obtained in single-DNA manipulation experiments. In this article, we analyze recent progress on theoretical approaches aimed at the construction of such a framework and discuss possible practical applications of the reviewed methods.

## 2. Conformational Energy of DNA

In order to describe the DNA behavior under mechanical constraints, it is important to know how the total energy of DNA depends on its global conformation. Numerous existing theoretical and experimental studies suggest that the mechanical properties of DNA in the general case can be very well approximated by the thin rod elastic theory [30,48,49,50,51,52,53]. Under this approximation, the bending, $E_{\mathrm{bend}}$, and twisting, $E_{\mathrm{twist}}$, elastic energies of a short DNA fragment of length *b* are: $E_{\mathrm{bend}}=\frac{L_{p}}{2b}\theta^{2}$ and $E_{\mathrm{twist}}=\frac{L_{\mathrm{tw}}}{2b}\varphi^{2}$. Here and below, all energies are presented in $k_{B}T$ units, where $k_{B}$ is the Boltzmann constant and *T* is the temperature of the surrounding environment. In the above formulas, *θ* and *φ* are the bending and twisting angles of the short DNA fragment, respectively. As for the parameters $L_{p}$ and $L_{\mathrm{tw}}$, which have a dimension of length (due to the energy normalization by $k_{B}T$), they are often referred to as the bending and twisting persistence lengths of DNA. These two parameters describe the bending and twisting rigidities of DNA; the larger the values of $L_{p}$ and $L_{\mathrm{tw}}$, the stiffer the DNA is. Finally, the elastic stretching deformation of DNA is typically neglected in theoretical studies, as it has been previously shown that even under very high tensions (50–60 pN), the relative change of the DNA contour length is <5% [54,55], which contributes to less than 5% of the conformational energy of the DNA. Therefore, the theoretical framework discussed in this review is mainly based on the inextensible semi-flexible polymer model of DNA.

It should be noted that the above approximations of the DNA bending and twisting elastic energies work well only when $b\ll L_{p}$ and $b\ll L_{\mathrm{tw}}$, so that the thermally-excited bend and twist of the short DNA fragment are sufficiently small (i.e., θ≪1 and φ≪1). In this case, it is not hard to show that $\hat{t}\cdot {\hat{t}}^{\prime }=\cos\theta \approx 1-\frac{1}{2}\theta^{2}$, where $\hat{t}$ and ${\hat{t}}^{\prime }$ are the unit vectors tangent to the DNA fragment at its ends. With this small note, the bending energy of the DNA fragment can be re-written in a more convenient form as $E_{\mathrm{bend}}=\frac{L_{p}}{2b}{\left(\hat{t}-{\hat{t}}^{\prime }\right)}^{2}$.

**Figure 1:**
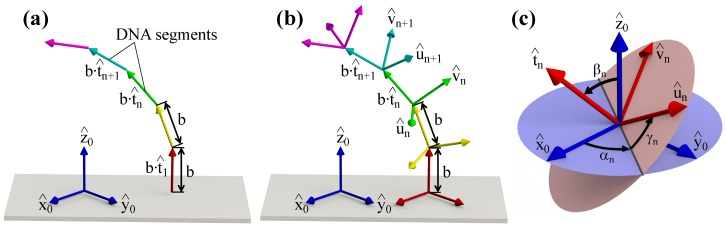
Discretized model of DNA. In theoretical computations, the DNA polymer is usually represented by a polygonal chain comprised of short DNA segments of equal length, *b*. While in the case of torsion-free DNA, the global DNA conformation is completely specified by the set of unit tangent vectors $\left({\hat{t}}_{1},...,{\hat{t}}_{N}\right)$ of the respective DNA segments (**a**). In a more general scenario, one needs to know the 3D-orientations of all of the DNA segments, which are treated as solid rigid bodies with attached local Cartesian coordinate frames $\left({\hat{t}}_{n},{\hat{u}}_{n},{\hat{v}}_{n}\right)$ (**b**). (**c**) Schematic picture of the three Euler rotation angles $\left(\alpha_{n},\beta_{n},\gamma_{n}\right)$ determining the orientation of the *n*-th DNA segment with respect to the fixed global coordinate system $\left({\hat{x}}_{0}^{},{\hat{y}}_{0}^{},{\hat{z}}_{0}^{}\right)$.

Using the above formulas for short DNA fragments, one can easily derive an expression for the conformational energy, $E_{\mathrm{conf}}$, of an arbitrarily long DNA. Indeed, by dividing the DNA polymer into small straight segments of equal length *b*, which form a polygonal chain representation of the DNA (see Figure 1a), the conformational energy can be simply found as the sum of local elastic bending and twisting energies:

(1) $$E_{\mathrm{conf}}=\sum_{n=1}^{N-1}\frac{L_{p}}{2b}{\left({\hat{t}}_{n+1}-{\hat{t}}_{n}\right)}^{2}+\sum_{n=1}^{N}\frac{L_{\mathrm{tw}}}{2b}\varphi_{n}^{2}$$

where *N* is the total number of DNA segments in the polygonal chain representing DNA; ${\hat{t}}_{n}$ and $\varphi_{n}$ are the tangent vector and the twisting angle of the *n*-th DNA fragment, respectively.

In the continuous limit, b→0, the above sums transform into two very well-known integrals from the elastic theory of thin rods:

(2) $$E_{\mathrm{conf}}=\frac{L_{p}}{2}\int_{0}^{L}\;{\left(\;\frac{d\hat{t}\left(s\right)}{ds}\;\right)}^{\;\;2}ds+\frac{L_{\mathrm{tw}}}{2}\int_{0}^{L}\;{\left(\;\frac{d\varphi (s)}{ds}\;\right)}^{\;\;2}ds$$

Here, *L* is the DNA contour length; $\hat{t}\left(s\right)$ and φ(s) are the tangent vector and the twist of the DNA polymer at the point situated on the DNA contour that corresponds to the arc length *s*.

While Equations (1) and (2) describe the elastic energy of unconstrained DNA, for practical use, it is frequently important to know how DNA behaves under force, $\hat{f}$, and torque, *τ*. The application of these mechanical constraints to DNA results in the appearance of two additional energy terms, $-\hat{f}\cdot \hat{d}$ and −2πτΔLk, in the expression for the total conformational energy of the DNA:

(3) $$E_{\mathrm{tot}}=E_{\mathrm{conf}}-\hat{f}\cdot \hat{d}-2\pi \tau \Delta \mathrm{Lk}$$

Here, $\hat{d}$ is the DNA end-to-end distance vector (i.e., $\hat{d}=b\sum_{n=1}^{N}{\hat{t}}_{n}$) and ΔLk is the DNA linking number change with respect to the torsionally-relaxed DNA state (see more details in Section 4). In this formula, the force $\hat{f}$ and torque *τ* are scaled by $k_{B}T$; thus, $\hat{f}$ has a dimension of length${}^{-1}$, and *τ* is dimensionless.

Knowing the total energy, it is possible to predict the DNA behavior in the thermodynamic equilibrium since it is completely determined by the Boltzmann distribution, $\frac{1}{Z}e^{-E_{\mathrm{tot}}}$. However, calculation of the DNA partition function, *Z*, in the general case can be extremely difficult. In the following sections, we review several theoretical approaches that have been developed to predict the equilibrium DNA behavior under force and torque constraints and discuss their potential applications.

## 3. Mechanically-Stretched Homogeneous DNA

In the simplest case of mechanically-stretched torsion-free homogeneous DNA, the above equations reduce to the so-called worm-like chain polymer model (WLC), and the total energy of DNA becomes [48]:

(4) $$E_{\mathrm{tot}}=\frac{L_{p}}{2}\int_{0}^{L}\;{\left(\;\frac{d\hat{t}\left(s\right)}{ds}\;\right)}^{\;\;2}ds-\hat{f}\cdot \hat{d}$$

To quantify the net average effect of the applied tension on the DNA behavior, the force-extension curve of DNA, $z\left(f\right)=\langle \hat{d}\cdot {\hat{z}}_{0}\rangle$, is typically measured in single-molecule experiments [54,56]. Here, *z* is the average DNA extension along the force direction described by the unit vector ${\hat{z}}_{0}$; $f=|\hat{f}|=\hat{f}\cdot {\hat{z}}_{0}$ is the force magnitude; and the averaging $\langle ...\rangle$ is performed over all of the equilibrium DNA conformations weighted by the corresponding Boltzmann factor, $\frac{1}{Z}e^{-E_{\mathrm{tot}}}$.

Detailed analysis of the above formula shows that the bending persistence length, $L_{p}$, separates the force-response of DNA into a low ($f\ll \frac{1}{L_{p}}$) and a high ($f\gg \frac{1}{L_{p}}$) force regime. In the low force regime, the DNA force-extension curve can be well approximated by the freely-jointed chain polymer (FJC) model: $L_{p}f=\frac{3}{2}\frac{z}{L}$; whereas in the high force regime, it exhibits highly non-linear behavior: $L_{p}f={\left[4{\left(1-\frac{z}{L}\right)}^{2}\right]}^{-1}$ [48,49]. A simple interpolation between the two regimes leads to the famous Marko–Siggia’s formula that has been shown to be able to describe the DNA behavior over the entire force range [48]:

(5) $$f=\frac{1}{L_{p}}\left[\frac{z}{L}+\frac{1}{4{\left(1-\frac{z}{L}\right)}^{2}}-\frac{1}{4}\right]$$

The simple yet very useful Marko–Siggia formula that relates the DNA extension change to the applied force has found many applications in the analysis of single-molecule experimental data over the ensuing years. However, the requirement of the DNA homogeneity makes it difficult to use this formula in situations where DNA inhomogeneities naturally arise as a result of sequence-dependent properties of DNA [6,57,58,59,60,61] or due to proteins binding to various DNA sites. The solution to this problem requires the construction of new approaches capable of taking into account local variations in the DNA structure.

## 4. Homogeneous DNA Subjected to Both Force and Torque Constraints

In the case of DNA subjected to both force and torque constraints, the total energy of DNA becomes:

(6) $$E_{\mathrm{tot}}=\frac{L_{p}}{2}\int_{0}^{L}\;{\left(\;\frac{d\hat{t}\left(s\right)}{ds}\;\right)}^{\;\;2}ds+\frac{L_{\mathrm{tw}}}{2}\int_{0}^{L}\;{\left(\;2\pi \frac{d\mathrm{Tw}(s)}{ds}\;\right)}^{\;\;2}ds-\hat{f}\cdot \hat{d}-2\pi \tau \Delta \mathrm{Lk}$$

Here, $\mathrm{Tw}\left(s\right)=\frac{1}{2\pi }\varphi \left(s\right)$ is the DNA twist number at the point corresponding to the arc length *s*, and ΔLk is the DNA linking number change with respect to the torsionally-relaxed DNA reference state.

By definition, the linking number, Lk, represents the number of times the two strands of DNA are twisted around each other [62,63,64,65,66,67]. Thus, knowing the architecture of the DNA duplex, it is easy to find the linking number of any torsion-free DNA structure as the ratio of the total DNA length to the helical repeat of the respective DNA form. For example, the helical repeat of DNA in B-form is $h_{B}=10.4$ base-pairs per turn. Hence, the linking number of a torsion-free B-DNA comprised of $N_{\mathrm{bp}}$ base-pairs is: ${\mathrm{Lk}}_{0,B}=N_{\mathrm{bp}}/h_{B}$.

Since DNA predominantly stays in B-form under physiological conditions, it is typically used as a reference state for all of the calculations based on Equation (6) with the DNA linking number change defined as: $\Delta \mathrm{Lk}=\mathrm{Lk}-{\mathrm{Lk}}_{0,B}$. It should be noted that at a fixed force, $\hat{f}$, and torque, *τ*, applied to DNA, its average linking number $\langle \mathrm{Lk}\rangle$ is simply proportional to the DNA length. Due to this reason, it is frequently more convenient to express the level of DNA supercoiling in terms of the DNA length-independent quantity $\sigma =\Delta \mathrm{Lk}/{\mathrm{Lk}}_{0,B}$, which is usually referred to as the superhelical density/linking number density/specific linking difference. Negative/positive value of *σ* corresponds to negatively (underwound) or positively (overwound) supercoiled DNA, respectively. This number shows how many turns are removed from/added to the DNA per a single turn of the two strands in the relaxed configuration.

Using Călugăreanu–White’s theorem [62,63], the DNA linking number change, ΔLk, can be conveniently expressed as a sum of two components: ΔLk=Tw+Wr, where Tw and Wr are the DNA twist and writhe numbers:

(7) $$\begin{array}{cc} & \mathrm{Tw}=\int_{0}^{L}\frac{d\mathrm{Tw}(s)}{ds}\;ds=\frac{1}{2\pi }\int_{0}^{L}\;\left[\hat{u}\left(s\right)\times \frac{d\hat{u}\left(s\right)}{ds}\right]\cdot \hat{t}\left(s\right)\;ds\\ & \mathrm{Wr}=\frac{1}{4\pi }\int_{0}^{L}\;\;\int_{0}^{L}\;\left[\hat{t}\left(s\right)\times \hat{t}\left(s^{\prime }\right)\right]\cdot \frac{\hat{r}\left(s\right)-\hat{r}\left(s^{\prime }\right)}{|\;\hat{r}\left(s\right)-\hat{r}\left(s^{\prime }\right){|}^{3}}\;ds\;ds^{\prime } \end{array}$$

Here, $\hat{r}\;\left(s\right)$ and $\hat{r}\;\left(s^{\prime }\right)$ are the position vectors of points situated on the DNA polymer that correspond to the arc lengths *s* and $s^{\prime }$, respectively; $\hat{t}\left(s\right)$ is the unit tangent vector, and $\hat{u}\left(s\right)$ is the unit vector normal to the DNA contour at the point *s*, such that $\hat{u}\left(s\right)$ keeps track of the DNA twist, Tw(s).

Due to the double-contour integral in the above expression for the DNA writhe number, it is clear that in general, Wr depends on the global DNA conformation. However, it is usually more convenient to use a simpler Fuller’s formula [67], which provides accurate estimates of the DNA writhe number for certain cases described below:

(8) $${\mathrm{Wr}}^{F}=\frac{1}{2\pi }\int_{0}^{L}\;\frac{\left[\hat{t}\left(s\right)\times \frac{d\hat{t}\left(s\right)}{ds}\right]\cdot {\hat{z}}_{0}^{}}{1+\hat{t}\left(s\right)\cdot {\hat{z}}_{0}^{}}\;ds$$

where, as before, ${\hat{z}}_{0}$ is the unit vector pointing in the direction of force $\hat{f}$ applied to the DNA. It can be shown that ${\mathrm{Wr}}^{F}=\mathrm{Wr}$ for those DNA conformations that can be obtained by a continuous deformation of DNA initially extended along the ${\hat{z}}_{0}$-axis direction in such a way that none of the intermediate DNA conformations are represented by a self-intersecting curve or a curve that has points at which the denominator of the integrand in Equation (8) resets to zero [50,67]. For example, Fuller’s formula accurately describes the writhe number of nearly extended or solenoid-shaped DNA conformations [51,52]. Thus, it can be used to predict the behavior of DNA under a wide range of mechanical constraints up to the onset of the torque-induced buckling transition when DNA starts to develop plectonemes [50,51,52,53,68,69].

While Equations (6) and (7) provide a general analytical background for theoretical studies of supercoiled DNA, in real calculations, it is often more convenient to use discretized versions of these formulas where DNA is modeled as a polygonal chain consisting of short segments of equal length *b* ($b\ll L_{p}$ and $b\ll L_{\mathrm{tw}}$); see Figure 1b. The orientation of each DNA segment in such a model is represented by a local Cartesian coordinate frame $\left({\hat{u}}_{n},{\hat{v}}_{n},{\hat{t}}_{n}\right)$ obtained by a three-dimensional rotation about the origin of a fixed lab coordinate system $\left({\hat{x}}_{0},{\hat{y}}_{0},{\hat{z}}_{0}\right)$: ${\hat{u}}_{n}=R_{n}{\hat{x}}_{0}$, ${\hat{v}}_{n}=R_{n}{\hat{y}}_{0}$, and ${\hat{t}}_{n}=R_{n}{\hat{z}}_{0}$. Here, *n* is the index of the corresponding DNA segment, and $R_{n}=R_{\alpha_{n}}R_{\beta_{n}}R_{\gamma_{n}}$ denotes a rotation matrix resulting from the composition of three successive Euler revolutions through angles $\alpha_{n}$, $\beta_{n}$ and $\gamma_{n}$ shown in Figure 1c (where $\alpha_{n},\gamma_{n}\in \left[0,2\pi \right]$ and $\beta_{n}\in \left[0,\pi \right]$) [69]. Since the Cartesian coordinate frame of each DNA segment $\left({\hat{u}}_{n},{\hat{v}}_{n},{\hat{t}}_{n}\right)$ is in a one-to-one relation with the respective rotational matrix $R_{n}$, hereafter, we simply use $R_{n}$ to designate the *n*-th DNA segment orientation.

**Figure 2:**
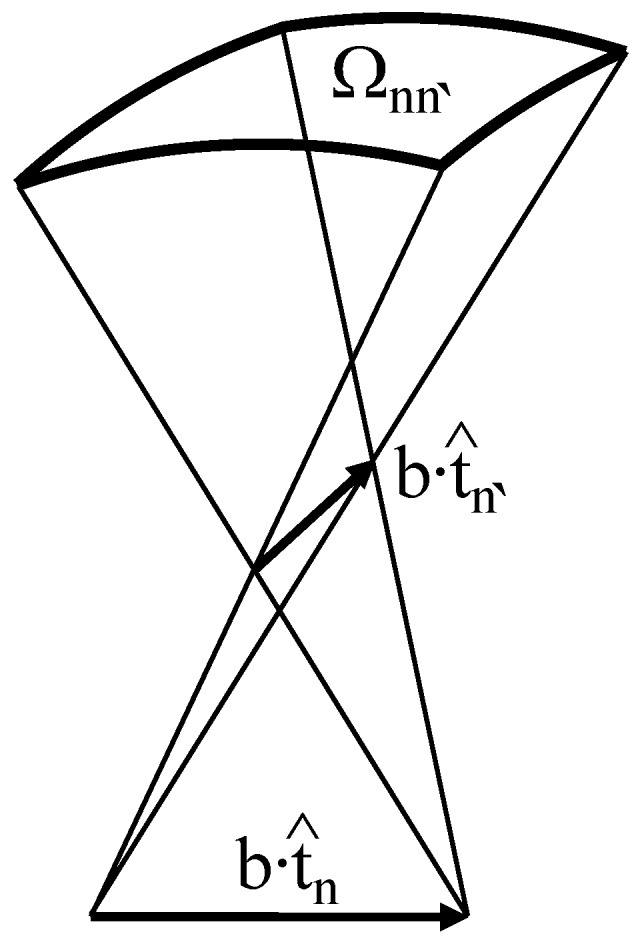
Solid angle $\Omega_{nn^{\prime }}$ corresponding to the quadrangle formed by the *n*-th and $n^{\prime }$-th DNA segments.

Using the polygonal chain representation of DNA, it can be shown that Equation (7) can be re-written in the following discretized form [70,71]:

(9) $$\begin{array}{cc} & \mathrm{Tw}=\sum_{n=1}^{N-1}\Delta {\mathrm{Tw}}_{n}\;\left(R_{n},R_{n+1}\right)\\ & \mathrm{Wr}=\frac{1}{4\pi }\sum_{\begin{array}{c} n,n^{\prime }=1\\ n\neq n^{\prime } \end{array}}^{N}\;\;\Omega_{nn^{\prime }} \end{array}$$

where $\Delta {\mathrm{Tw}}_{n}\left(R_{n},R_{n+1}\right)=\frac{1}{2\pi }\;\left[{\hat{u}}_{n}\times {\hat{u}}_{n+1}\right]\cdot {\hat{t}}_{n}=\frac{1}{2\pi }$ $\left[R_{n}{\hat{x}}_{0}\times R_{n+1}{\hat{x}}_{0}\right]\cdot R_{n}{\hat{z}}_{0}$ is the twist number corresponding to the local contribution of the *n*-th and (n+1)-th DNA segments, which is simply the twist angle, $\varphi_{n}$, between the coordinate systems described by rotation matrices $R_{n}$ and $R_{n+1}$ normalized to 2π; and $\Omega_{nn^{\prime }}$ is the solid angle corresponding to the quadrangle formed by the *n*-th and $n^{\prime }$-th DNA segments (see Figure 2), which can be found by using Equations (14)–(16) from [71].

Combining together Equations (1), (3) and (9) and taking into account that $\hat{f}\cdot \hat{d}=\hat{f}\cdot b\sum_{n=1}^{N}{\hat{t}}_{n}=bf\sum_{n=1}^{N}{\hat{z}}_{0}\cdot R_{n}{\hat{z}}_{0}$, it is straightforward to obtain the discretized version for the total conformational energy of DNA:

(10) $$\begin{array}{c} E_{\mathrm{tot}}\;\left(R_{1},...,R_{N}\right)=\sum_{n=1}^{N-1}\frac{L_{p}}{2b}{\left[R_{n+1}{\hat{z}}_{0}^{}-R_{n}{\hat{z}}_{0}^{}\right]}^{2}+\sum_{n=1}^{N-1}\frac{L_{\mathrm{tw}}}{2b}{\left[2\pi \Delta {\mathrm{Tw}}_{n}\;\left(R_{n},R_{n+1}\right)\right]}^{2}\\ -bf\sum_{n=1}^{N}{\hat{z}}_{0}^{}\cdot R_{n}{\hat{z}}_{0}^{}-2\pi \tau \left[\sum_{n=1}^{N-1}\Delta {\mathrm{Tw}}_{n}\;\left(R_{n},R_{n+1}\right)+\frac{1}{4\pi }\sum_{\begin{array}{c} n,n^{\prime }=1\\ n\neq n^{\prime } \end{array}}^{N}\;\;\Omega_{nn^{\prime }}\right] \end{array}$$

## 5. Metropolis-Monte Carlo Simulations

Having at hand the formula for the total conformational energy of DNA (Equation (10)), one can apply the Metropolis-Monte Carlo algorithm [72,73] to computationally generate equilibrium DNA conformations that satisfy the Boltzmann distribution [74,75]. By getting a sufficiently large number of such DNA states, it is then possible to predict how various observable DNA characteristics, including the average DNA extension and linking number change, evolve in response to mechanical constraints applied to the DNA.

**Figure 3:**
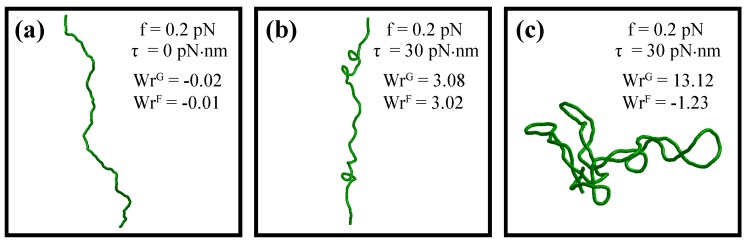
Examples of DNA equilibrium conformations obtained by the Metropolis-Monte Carlo algorithm. In the absence of torque, mechanically-stretched DNA typically assumes an extended chain configuration (**a**). On the other hand, the application of sufficiently large positive or negative torque leads to the DNA buckling transition (**b**), which eventually results in the formation of supercoiled DNA plectoneme structures (**c**). Evaluation of the DNA writhe number by the Gauss double contour integral (${\mathrm{Wr}}^{G}$) and Fuller’s formula (${\mathrm{Wr}}^{F}$) shows that both formulas predict very similar values of the DNA writhe number up to the onset of the DNA buckling transition. For plectonemic DNA structures, however, Fuller’s formula does not provide accurate estimations. In the above simulations, the length of DNA was set to 0.85 μm.

Figure 3 shows examples of representative polymer conformations obtained via the Metropolis-Monte Carlo algorithm for mechanically-stretched DNA at two different torques. As can be seen from the figure, in the case of torsionally-relaxed DNA (τ=0), the polymer typically assumes an extended conformation, which does not show preference for the formation of either positively- or negatively-coiled DNA structures; see Figure 3a. Upon the increase of the torque magnitude, the DNA undergoes the so-called buckling transition, as shown in Figure 3b, featured by the formation of multiple DNA writhes, which eventually develop into supercoiled DNA plectonemes; see Figure 3c.

It is interesting to note that the estimations of the DNA writhe number by the Gauss double contour integral, ${\mathrm{Wr}}^{G}$ (Equation (7)) and Fuller’s formula, ${\mathrm{Wr}}^{F}$ (Equation (8)) agree with each other very well up to the onset of the DNA buckling transition, in full accordance with the previously published theoretical studies [50,51,52,53]. However, as soon as DNA starts to form supercoiled plectonemes, the writhe number predicted by Fuller’s formula starts to deviate from the one computed from the Gauss double contour integral. Thus, any interpretations of the observed DNA behavior involving Fuller’s formula, which is frequently used in theoretical studies of DNA, should be restricted up to the onset of the DNA buckling transition [50,51].

Metropolis-Monte Carlo simulations have been widely used to investigate the response of DNA to various mechanical constraints, providing valuable quantitative description and interpretation of numerous single-DNA manipulation experiments [52,53,74,75,76]. However, in order to obtain sufficiently large and accurate Boltzmann statistics of equilibrium DNA states, a considerable number of the algorithm iterations have to be carried out, which typically requires extensive computational resources. Together with the lack of detailed analytical insights into the DNA behavior, this imposes significant restrictions on the predictive power of such a numerical approach.

## 6. Transfer-Matrix Formalism

### 6.1. Transfer-Matrix Calculations of the DNA Partition Function

In order to gain more detailed understanding of the DNA micromechanics under force and torque constraints, several analytic polymer theories have been developed during recent years [12,50,51,52,53,76,77,78]. While these studies resulted in many important insights into the equilibrium behavior of DNA, most of them were mainly based on the assumption of the uniformity of the DNA physical properties without taking into account possible local variations, which may either come from the sequence-dependence of the DNA parameters, local DNA transitions into alternative structural states or due to proteins binding to various DNA sites.

In contrast to the above theories, the recently-described transfer-matrix formalism allows one to take into consideration both local deformations and structural transformations of DNA [68,69,79,80,81]. The main idea of the method is based on the nearest neighbor approximation. It has been shown that if the total energy of DNA can be written as the sum of local DNA segments contributions, $E_{\mathrm{tot}}=\sum_{n=1}^{N-1}E_{n}\left(R_{n},R_{n+1}\right)$, then the DNA partition function, *Z*, can be found as a trace of the ordered product of transfer-matrices $S_{n}$ defined on the vertices that join neighboring segments in the discretized DNA model: $Z∼\mathrm{Tr}(\prod_{n=1}^{N-1}S_{n})$; see [68,69,79,80] and the details below. Here, $E_{n}\left(R_{n},R_{n+1}\right)$ is the energy contribution corresponding to the vertex connecting the *n*-th and (n+1)-th segments of the polygonal chain representing the DNA polymer. As matrices $S_{n}$ depend only on the local physical properties of the respective DNA segments, it is clear that the transfer-matrix calculations do not require the assumption of the DNA homogeneity.

Because of these advantages, the transfer-matrix approach may provide a wide scope of potential applications, such as the investigation of sequence-dependent DNA response to mechanical constraints and the exploration of the interactions between DNA and architectural proteins. However, the requirement of the DNA total conformational energy being expressed as the sum of local DNA segments contributions imposes certain limits on the transfer-matrix method applications in the case of DNA subjected to torque constraints.

Indeed, as was mentioned in Section 4, the DNA writhe number generally depends on the global DNA conformation and cannot be written as a simple sum of local DNA segments’ contributions; see Equation (9). This limitation can be partially overcome by utilizing Fuller’s formula (Equation (8)), which provides correct estimates of the DNA writhe number up to the onset of the torque-induced buckling transition when DNA starts to develop supercoiled plectonemes; see [51,52,53] and Figure 3. Although the application of Fuller’s formula beyond the DNA buckling transition point results in inaccurate evaluation of the DNA writhe number (see Figure 3c), it has been shown in our recent work that with a slight revision of the expression for the DNA total conformational energy, the transfer-matrix formalism still can be used to predict the DNA behavior in a wide range of force and torque constraints [69]. Namely, by introducing an additional energy term, $\tau \lambda {\mathrm{Wr}}^{F}$, with a free model parameter, *λ*, it is possible to describe the force- and torque-response of DNA up to the experimentally-measured DNA buckling transition boundary, which can be reproduced in the transfer-matrix calculations simply by tuning the value of *λ*.

Taking into account the above notes and introducing the new energy term $\tau \lambda {\mathrm{Wr}}^{F}=\tau \lambda \left(\Delta {\mathrm{Lk}}^{F}-\mathrm{Tw}\right)$ into Equation (10), it can be shown that the total conformational energy of DNA can be expressed with the help of Fuller’s formula as [69]:

(11) $$E_{\mathrm{tot}}\;\left(R_{1},...,R_{N}\right)=\sum_{n=1}^{N-1}\;E_{n}\;\left(R_{n},R_{n+1}\right)-bf\left({\hat{z}}_{0}^{}\cdot R_{N}{\hat{z}}_{0}^{}\right)$$

where the local energy terms corresponding to neighboring DNA segments contributions assume the following form:

(12) $$E_{n}\;\left(R_{n},R_{n+1}\right)=\frac{a}{2}{\left(R_{n}{\hat{z}}_{0}^{}\;-\;R_{n+1}{\hat{z}}_{0}^{}\right)}^{2}+\frac{c}{2}{\left(2\pi \Delta {\mathrm{Tw}}_{n}\right)}^{2}-bf\left({\hat{z}}_{0}^{}\cdot R_{n}{\hat{z}}_{0}^{}\right)-\tau \left(2\pi \;-\;\lambda \right)\Delta {\mathrm{Lk}}_{n}^{F}-\tau \lambda \Delta {\mathrm{Tw}}_{n}$$

Here, $a=L_{p}/b$ and $c=L_{\mathrm{tw}}/b$ are dimensionless parameters describing the bending and twisting rigidities of DNA segments in the discretized model; $\Delta {\mathrm{Tw}}_{n}=\Delta {\mathrm{Tw}}_{n}\left(R_{n},R_{n+1}\right)$ and $\Delta {\mathrm{Lk}}_{n}^{F}=\frac{1}{2\pi }\left({\tilde{\alpha }}_{n+1}\;+\;{\tilde{\gamma }}_{n+1}\;-\;{\tilde{\alpha }}_{n}\;-\;{\tilde{\gamma }}_{n}\right)$ are local DNA segments contributions to the total DNA twist and linking number change, respectively [50,69], where ${\tilde{\alpha }}_{n}$ and ${\tilde{\gamma }}_{n}$ are the Euler angles of the *n*-th DNA segment from the extended range of (−∞,+∞).

Knowing the total conformational energy of DNA under tension, *f*, and torque, *τ*, its partition function, $Z_{f,\tau }$, can be found as:

(13) $$Z_{f,\tau }=\int \;\;dR_{1}...dR_{N}\;e^{-E_{\mathrm{tot}}\left(R_{1},...,R_{N}\right)}\xi \;\left(R_{N},R_{1}\right)=\int \;\;dR_{1}...dR_{N}\;\prod_{n=1}^{N-1}T\;\left(R_{n},R_{n+1}\right)\times \sigma \;\left(R_{N},R_{1}\right)$$

where $\xi (R_{N},R_{1})$ is a function that imposes specific boundary conditions on the orientations of the DNA ends; $\sigma \left(R_{N},R_{1}\right)=\xi \left(R_{N},R_{1}\right)e^{bf({\hat{z}}_{0}\cdot R_{N}{\hat{z}}_{0})}$ and $T\left(R_{n},R_{n+1}\right)=e^{-E_{n}\left(R_{n},R_{n+1}\right)}$. In the above formula, the integration is carried out over all of the possible DNA segments’ orientations, i.e., $\int \;dR_{n}=\int_{0}^{2\pi }\;d\alpha_{n}\int_{0}^{2\pi }\;d\gamma_{n}\int_{0}^{\pi }\;\sin\beta_{n}\;d\beta_{n}$.

Equation (13) can be further simplified by recalling that any square-integrable function, *ψ*, defined on the SO(3) group of 3D rotation matrices parametrized by Euler angles α,β,γ can be expanded into a series of orthogonal D-functions, $D_{ml}^{n}\left(\alpha ,\beta ,\gamma \right)$ [82]. The expansion coefficients of this series can be found as $\psi_{mln}=\frac{2n+1}{8\pi^{2}}\int \;dR\;\psi \left(R\right){\bar{D}}_{ml}^{n}\left(R\right)$, where for the sake of simplicity, we use the following notations: ψ(R)=ψ(α,β,γ) and $D_{ml}^{n}\left(R\right)=D_{ml}^{n}\left(\alpha ,\beta ,\gamma \right)$. The bar over function $D_{ml}^{n}$ in the above formula denotes complex conjugation. Performing such an expansion for functions $\sigma (R_{N},R_{1})$ and $T(R_{n},R_{n+1})$, it can be shown that for a sufficiently long DNA ($L\gg L_{p}$ and $L\gg L_{\mathrm{tw}}$), Equation (13) reduces to a mere multiplication of matrices whose entries are the expansion coefficients of $T(R_{n},R_{n+1})$ and $\sigma (R_{N},R_{1})$ functions (see [69] for more details):

(14) $$Z_{f,\tau }=\mathrm{Tr}\left(S^{N\;-\;1}V\right)$$

where the elements of matrices *S* and *V* are:

(15) $$\begin{array}{cc} \;\;S_{kk^{\prime }}\;\left(a,b,c,\lambda \right)=\; & \pi^{2}\sqrt{\left(2k\;+\;1\right)\left(2k^{\prime }\;\;+\;1\right)}\;e^{-a-c}\;\\ & \times \;\;\sum_{p,s,s^{\prime }\;,r}\;\;\left(2p\;+\;1\right)\left(2s\;+\;1\right)\left(2s^{\prime }\;\;+\;1\right)e^{ir(\omega -\frac{\pi }{2})}I_{r}\left(\tau \left[1\;-\;\frac{\lambda }{2\pi }\right]\right)I_{r}\left(c\sqrt{1\;+\;\chi^{2}}\;\right)\\ & \times L_{r}^{p}\left(-a\right)L_{r}^{s}\left(-bf\right)L_{r}^{s^{\prime }}\;\left(0\right)\;{\left(\;\;\begin{array}{ccc} p & s & k\\ -r & r & 0 \end{array}\;\right)}^{\;\;2}\;\;{\left(\;\;\begin{array}{ccc} p & s^{\prime } & k^{\prime }\\ -r & r & 0 \end{array}\;\right)}^{\;\;2} \end{array}$$

and:

(16) $$V_{kk^{\prime }}\left(b\right)=8\pi^{2}\delta_{k^{\prime }0}\;i_{k}\left(bf\right)\sqrt{2k\;+\;1}$$

Here, *i* is the imaginary unit; $\chi =\frac{\tau \lambda }{2\pi c}$ and $\omega =\tan^{-1}\left(\chi \right)$; $I_{r}\left(x\right)$ are modified Bessel functions of the first kind; $i_{k}\left(x\right)$ are spherical modified Bessel functions of the first kind; $L_{r}^{k}\left(s\right)=\int_{-1}^{\;1}d_{rr}^{k}\left(\cos^{-1}x\right)e^{-sx}dx$ is the bilateral Laplace transform of the diagonal elements, $d_{rr}^{k}$, of the Wigner small d-matrix; and $\left(\begin{array}{ccc} j_{1} & j_{2} & j_{3}\\ m_{1} & m_{2} & m_{3} \end{array}\right)$ are the Wigner 3-j symbols. Note that the parallel boundary condition (${\hat{t}}_{1}={\hat{t}}_{N}={\hat{z}}_{0}$), which was originally used to derive Equations (15) and (16), does not affect the final results as long as the DNA contour length is much larger than the DNA bending and twisting persistence lengths ($L\gg L_{p}$ and $L\gg L_{\mathrm{tw}}$) [69].

Knowing the partition function $Z_{f,\tau }$, it is then straightforward to calculate the DNA force-extension curve under a constant torque ($\tau =\tau_{0}$) and/or the torque-extension curve under a constant force ($f=f_{0}$) as: $z_{\tau_{0}}\left(f\right)=\frac{\partial \ln(Z_{f,\tau =\tau_{0}})}{\partial f}$ and $z_{f_{0}}\left(\tau \right)=\frac{\partial \ln(Z_{f,\tau })}{\partial f}{|}_{f=f_{0}}$, respectively. The corresponding DNA linking number changes under the above mechanical constraints are: $\Delta Lk_{\tau_{0}}\left(f\right)=\frac{1}{2\pi }\frac{\partial \ln(Z_{f,\tau })}{\partial \tau }{|}_{\chi =\mathrm{const}}^{\tau =\tau_{0}}$ and $\Delta Lk_{f_{0}}\left(\tau \right)=\frac{1}{2\pi }\frac{\partial \ln(Z_{f=f_{0},\tau })}{\partial \tau }{|}_{\chi =\mathrm{const}}$.

Combining these formulas with Equations (14)–(16), it is possible to predict the DNA behavior in a wide range of mechanical constraints, assuming that DNA does not experience structural transitions between alternative forms [69]. However, as has been mentioned in the Introduction (Section 1), numerous experimental data show that DNA frequently undergoes structural transformations when it is subjected to sufficiently large force and/or torque. In the next two sections, we review the application of the transfer-matrix formalism to understanding the force- and torque-dependent stability of such DNA structures.

### 6.2. Description of the DNA Structural Transitions Using the Transfer-Matrix Formalism

From single-molecule experiments, it is known that double-stranded DNA can exist in several structural states, including B-, L-, P- and S-DNA, which have very different geometric characteristics and material properties [2,4,5,6,8,9,10,11,16,21,22,23,28,54,56]. Furthermore, it has been found that the relative stability of these structures sensitively depends on the mechanical constraints applied to DNA. Understanding of the physical mechanisms responsible for the observed force- and torque-induced DNA transitions between various DNA forms has imposed a huge theoretical challenge to the field.

Several alternative approaches have been developed so far to solve the above problem [12,69,76,78] with two of the most recent methods being based on the transfer-matrix calculations and the evaluation of the energy global minima state of various DNA structures [12,69]. While the latter approach rests on the assumption of DNA homogeneity similar to many previous theories, the transfer-matrix method is free from such limitation and, in fact, requires only minor changes in the above formulas in order to incorporate various DNA structures into the partition function computations.

Indeed, by taking B-DNA as the reference state, transitions from B- to L-, P- or S-DNA forms are determined by the energy difference between the respective DNA structures, which includes a contribution from the base-pairing energy difference, $\mu_{u}$, and the respective contributions from the applied force, $-b_{u}f\left({\hat{z}}_{0}\cdot R_{n}{\hat{z}}_{0}\right)$, and torque, $-2\pi \tau \Delta lk_{0}^{\left(u\right)}$. Here, u= L, P, S or B denotes the structural state of the DNA segments in the discretized model of DNA; $b_{u}$ is the length of DNA segments in the respective state, *u*; and $\Delta lk_{0}^{\left(u\right)}=lk_{0,u}\;-\;lk_{0,B}$, where $lk_{0,u}=\pm h_{u}^{-1}$ is the relaxed linking number of the respective DNA form per single base-pair, which is assigned to be positive for right-handed DNA helical structures (like B-, P- or S-DNA) and negative for left-handed structures (L-DNA). Since we use B-DNA as the reference point, it is clear that $\mu_{B}=0$ and $\Delta lk_{0}^{\left(B\right)}=0$.

Utilizing the above notations, the total conformational energy of DNA in the general case can be found as:

(17) $$E_{\mathrm{tot}}\;\left(u_{1},...,u_{N},R_{1},...,R_{N}\right)=\sum_{n=1}^{N-1}E_{n}^{\left(u_{n}\right)}\;\;\left(R_{n},R_{n+1}\right)+q\sum_{n=1}^{N}\left(\mu_{u_{n}}\;-\;2\pi \tau \Delta lk_{0}^{\left(u_{n}\right)}\right)-b_{u_{N}}f\;\left({\hat{z}}_{0}^{}\cdot R_{N}{\hat{z}}_{0}^{}\right)$$

where $\left(R_{1},...,R_{N}\right)$ and $\left(u_{1},...,u_{N}\right)$ are the orientations and structural states of DNA segments in the discretized model of DNA; *q* is the number of base-pairs in each segment; $b_{u}=q\rho_{u}$ is the length of the DNA segments in state *u*, where $\rho_{u}$ is the base-pair rise in the corresponding DNA structure. As for the local energy terms, $E_{n}^{\left(u_{n}\right)}\;\left(R_{n},R_{n+1}\right)$, they have absolutely the same form as in Equation (12) with the only difference being that parameters $a_{u}=L_{p}^{\left(u\right)}/b_{u}$, $c_{u}=L_{\mathrm{tw}}^{\left(u\right)}/b_{u}$ and $\lambda_{u}$ describing the respective physical properties of DNA segments now depend on the segments’ state, u= L, P, S or B. Here, $L_{p}^{\left(u\right)}$ and $L_{\mathrm{tw}}^{\left(u\right)}$ are the bending and twisting persistence lengths of DNA in state *u*. The last term in Equation (17) corresponding to the potential energy of the DNA end segment is negligible in the general case compared to the total conformational energy of DNA and is kept in Equation (17) solely for the purpose of mathematical rigorousness.

Since Equations (11) and (17) have a very similar appearance, it can be shown that the transfer matrix, $S_{u}$, for DNA segments being in state *u* can be obtained simply via multiplication of Equation (15) by the exponential factor $e^{-q(\mu_{u}\;-\;2\pi \tau \Delta lk_{0}^{\left(u\right)})}$ [69]:

(18) $${\left(S_{u}\right)}_{kk^{\prime }}=e^{-q\left(\mu_{u}-2\pi \tau \Delta lk_{0}^{\left(u\right)}\right)}S_{kk^{\prime }}\;\left(a_{u},\;b_{u},\;c_{u},\;\lambda_{u}\right)$$

Then, by using Equation (18), the DNA partition function in the general case can be found as (see [69] for more details):

(19) $$Z_{f,\tau }=\mathrm{Tr}\left(U{\hat{S}}^{N\;-\;1}\hat{V}\right)$$

where $U=\left(\begin{array}{cccc} I & I & I & I \end{array}\right)$ is a block-matrix comprising four identity matrices, *I*; and the transfer matrix, $\hat{S}$, and boundary condition matrix, $\hat{V}$, are:

(20) $$\;\;\;\;\hat{S}\;=\;\;\left(\;\;\begin{array}{cccc} S_{B} & S_{B} & S_{B} & S_{B}\\ S_{L} & S_{L} & S_{L} & S_{L}\\ S_{P} & S_{P} & S_{P} & S_{P}\\ S_{S} & S_{S} & S_{S} & S_{S} \end{array}\;\right)\;\;,\;\mathrm{and}\;\hat{V}\;\;=\;\;\left(\;\;\begin{array}{c} V_{\;B}\\ e^{-q\left(\mu_{L}-2\pi \tau \Delta lk_{0}^{\left(L\right)}\right)}V_{\;L}\\ e^{-q\left(\mu_{P}-2\pi \tau \Delta lk_{0}^{\left(P\right)}\right)}V_{\;P}\\ e^{-q\left(\mu_{S}-2\pi \tau \Delta lk_{0}^{\left(S\right)}\right)}V_{\;S} \end{array}\;\right)$$

Here, blocks $S_{u}$ are defined by Equation (18), and blocks $V_{u}=V\;\left(b_{u}\right)$ are described by the previously introduced Equation (16).

By performing differentiation of the DNA partition function defined by Equation (19) with respect to the force, *f*, or torque, *τ*, as was described at the end of Section 6.1, it is straightforward to calculate the DNA force-extension and/or the torque-extension curves under various mechanical constraints. The results of such computations, as well as their discussions can be found in the next section.

## 7. Force- and Torque-Dependent DNA Structural Transitions

The above transfer-matrix approach provides a general theoretical framework for analyzing data collected in single-DNA stretching and twisting experiments. To demonstrate its application, we calculated the force-extension curves, $z_{\tau_{0}}\left(f\right)$, and force-superhelical density curves, $\sigma_{\tau_{0}}\left(f\right)$, under a constant torque ($\tau =\tau_{0}$), as well as torque-extension curves, $z_{f_{0}}\left(\tau \right)$, and torque-superhelical density curves, $\sigma_{f_{0}}\left(\tau \right)$, under a constant force ($f=f_{0}$) constraints, for DNA that can switch between different structural forms. Here, we report results extending the previously published theoretical data from [69] to a larger force range by including S-DNA structural state in the transfer-matrix computations to make the physical picture more complete.

In all of our calculations, the size of the DNA segments was set to be equal to q=1.5 bp for all of the DNA structures. The rest of the parameters used in the transfer-matrix analysis are summarized in Table 1, with reference to the experimental and theoretical DNA studies from which the parameters’ values have been extracted.

**Figure 4:**
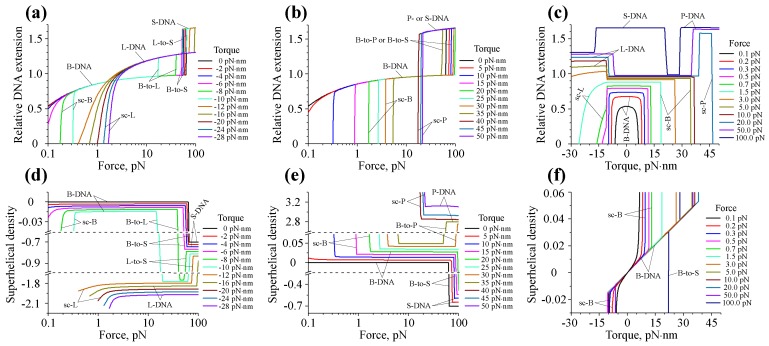
Structural stability of DNA under various mechanical constraints. (**a,b**) DNA force-extension curves, $z_{\tau_{0}}\left(f\right)$, and (**d,e**) force-superhelical density curves, $\sigma_{\tau_{0}}\left(f\right)$, calculated at different values of the applied torque ($\tau =\tau_{0}$). Panels (a,d) and (b,e) display results obtained for negative and positive torques, respectively. Abrupt changes in the behavior of the DNA force-extension curves presented on panels (a,b) mark the DNA transitions from B-form into alternative L-, P- or S-DNA structures, which are denoted by the B-to-L, B-to-P, B-to-S and L-to-S tags. The graphs on panels (d,e) show that these transitions are accompanied by a drastic change of the DNA superhelical density due to the large difference in the relaxed linking numbers of the corresponding DNA forms. (**c**) DNA torque-extension curves, $z_{f_{0}}\left(\tau \right)$, and (**f**) torque-superhelical density curves, $\sigma_{f_{0}}\left(\tau \right)$, at different values of the applied force ($f=f_{0}$). At $f_{0}<0.5$ pN, all torque-extension curves have symmetric profiles with respect to both positive and negative torques, while at larger forces ($f_{0}\geq 0.5$–0.7 pN), this symmetry breaks due to B-DNA switching into alternative L- and P-DNA structures. In panels (a–c), the DNA extension is normalized to the total contour length of DNA in B-form. The following abbreviations are used to indicate different DNA states and structural transitions in the above plots: sc-B, sc-L and sc-P, supercoiled B-, L- and P-DNA, respectively; B-to-L, B-to-P, B-to-S and L-to-S, structural transitions between the respective DNA forms.

As can be seen from Figure 4a,b,d,e, the DNA force-extension and force-superhelical density curves demonstrate two types of behavior: (a) smooth continuous variation with the applied force corresponding to the force-response of a particular DNA form; and (b) discontinuous, stepwise change, which indicates DNA transition from one structural state to another.

While at small torques DNA stays in the B-form and has a preferentially extended conformation, application of a moderate torsional stress to DNA results in the development of supercoiled structures (sc-B), whose formation is manifested by a rapid drop of the DNA extension and a corresponding change in the DNA superhelical density; see Figure 4a,b,d,e. A further increase of the torque magnitude leads to the B-to-L structural transition of DNA at high negative torques (≲−10 pN·nm) and B-to-P transition at high positive torques (≳35–40 pN·nm). Both of these DNA transformations are accompanied by a steep change in the DNA superhelical density seen on Figure 4d,e, which results from the very large difference in the relaxed linking numbers of B-, L- and P-DNA forms. Altogether, these data suggest that the structural state of DNA can be strongly modulated by mechanical constraints.

It is interesting to note that in contrast to B-to-L and B-to-P DNA transitions, which take place even at low DNA tensions, DNA switching from the B- to S-DNA form happens only when the force, *f*, exerted on the DNA has a sufficiently large magnitude (≥50 pN). Such a distinct behavior of L- and P-DNAs, on the one hand, and S-DNA, on the other, mainly originates from the large difference in the structure of the DNA duplex in these DNA forms. Due to rather small helical repeats ($h_{L}=16$ bp and $h_{P}=3$ bp), L- and P-DNA have much higher relaxed linking number densities ($\Delta {\mathrm{lk}}_{0,L}=-0.159$ and $\Delta {\mathrm{lk}}_{0,P}=0.237$) compared to that of S-DNA ($h_{S}=35$ bp and $\Delta {\mathrm{lk}}_{0,S}=-0.068$); see Table 1. This leads to strong stabilization of L- and P-DNA at large negative and positive torques, respectively, resulting in their dominance over the S-DNA form. In contrast, elongated S-DNA, whose contour length is ∼1.7-times larger than that of B-DNA, is more strongly stabilized by DNA stretching, explaining the occurrence of this DNA structure and the L-to-S transition at high forces.

Figure 4c,f showing torque-extension and torque-superhelical density curves reveal further details regarding the DNA structural transitions between different forms. It can be seen that in the case of $f_{0}\;<0.5$ pN, all $z_{f_{0}}\left(\tau \right)$ curves have symmetric profiles with respect to both positive and negative torques. However, as soon as the applied force increases above $f_{0}\;∼0.5$–0.7 pN, this symmetry breaks due to B-DNA switching into the L-DNA state at sufficiently large negative torques. A further increase of the applied force beyond $f_{0}∼15$ pN results in the appearance of the B-to-P transition, which takes place at ∼35–40 pN·nm torque.

**Figure 5:**
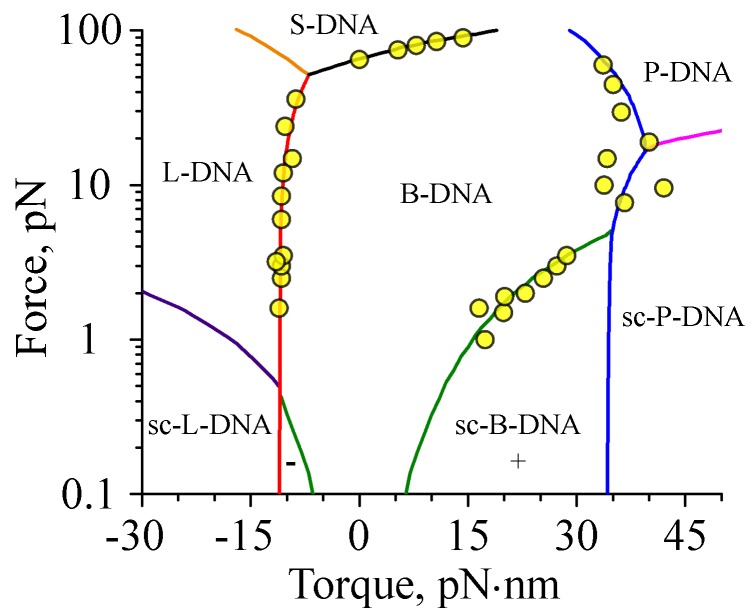
DNA phase diagram. Solid curves indicate transition boundaries between extended (B, L, P, S) and supercoiled (sc-B, sc-L, sc-P) states of DNA predicted by the transfer-matrix calculations. The resulting phase diagram demonstrates good agreement with the existing experimental data (circles), which were digitized from [4,5,6,8,10,11,22,23].

Using the above theoretical data, we plotted the DNA phase diagram showing the transition boundaries between different DNA states (see Figure 5). The supercoiling transition boundary for each DNA structure was assumed to pass through the points on the force-torque diagram where the DNA extension experiences an ∼50% drop with respect to the value predicted by the worm-like chain model for the corresponding DNA structure. As for the boundaries between alternative DNA forms (B, L, P or S), they were defined as the set of points $\left(f_{0},\tau_{0}\right)$ at which ∼50% of the DNA segments are in L-, P-, or S-DNA states, respectively. For this purpose, the total number of DNA segments, $N_{u}$, in state u= L, P or S at various values of the applied force $\left(f=f_{0}\right)$ and torque $\left(\tau =\tau_{0}\right)$ was calculated as ${\left(N_{u}\right)}_{f_{0},\tau_{0}}=-\frac{1}{q}\frac{\partial \ln(Z_{f,\tau })}{\partial \mu_{u}}{|}_{\tau =\tau_{0}}^{f=f_{0}}$.

As can be seen from Figure 5, the resulting phase diagram demonstrates good agreement with existing experimental data, suggesting that the transfer-matrix calculations correctly describe the experimentally observed behavior of DNA [4,5,6,8,10,11,22,23].

## 8. Other Potential Applications of the Transfer-Matrix Formalism

In this article, we reviewed several theoretical methods that can be used to investigate the DNA behavior under force and torque constraints, with a special focus on the powerful transfer-matrix approach whose application to the study of the DNA structural transitions has been demonstrated.

It should be noted that while we discussed only the DNA response to torsional stress, it is equally important to understand how DNA behaves when the DNA linking number is subjected to mechanical constraints instead of torque. This type of information may provide additional important insights into the physical processes underlying the DNA structural transitions, as has been demonstrated in a number of previously-reported theoretical studies [51,52,53,76,84,85,86,87]. By using a Dirac delta function approach similar to that described in [52,53,80], it is not hard to extend the transfer-matrix formalism to the fixed DNA linking number scenario, covering a wide range of single-DNA manipulation studies.

Besides the DNA structural transitions, the transfer-matrix calculations can also be employed to investigate DNA-protein interactions under various mechanical constraints. This can be done by treating the protein-bound DNA segments in the discretized polymer model as a new structural state, which is characterized by the protein binding energy, as well as the relaxed linking number change, and bending and twisting rigidities corresponding to the formed nucleoprotein complexes (see the details in [68,88,89] for the case of a mechanically-stretched torsion-free DNA interacting with small DNA-binding proteins). Such an approach allows one to apply the transfer-matrix formalism to gain valuable insights into physical processes governing DNA-protein interactions.

More importantly, this method can be used to solve the inverse problem, as well; by fitting the data collected in single-DNA manipulation experiments, one can obtain detailed information about the physical properties of nucleoprotein complexes formed on DNA. Indeed, upon binding to DNA, proteins often cause certain types of DNA deformations, such as bending or chiral wrapping, resulting in changes to the bending/twisting rigidities, as well as the relaxed linking number of the protein-bound DNA, which can be measured in experiments [31,32,33,34,35,36,37,38,39,40,41,42,68,90,91,92]. By fitting the experimental DNA force-extension and/or torque-extension data points to the theoretical curves predicted by the transfer-matrix calculations, one can determine the elastic properties of the formed nucleoprotein complexes and estimate the apparent equilibrium dissociation constant of the studied protein from DNA as a function of force and torque constraints exerted on the DNA.

Furthermore, the capability of the transfer-matrix calculations to take into account local sequence-dependent inhomogeneities of the DNA properties makes it possible to use this method to predict the average occupancies of various DNA sites by a protein that preferentially binds to certain sequence motifs in the context of DNA subjected to various mechanical constraints. Such an approach can be further extended to study competitive binding of multiple protein species to the same DNA and its potential modulation by DNA twisting and stretching. This type of theoretical research will be important in the near future as it may help to gain deeper understanding of the molecular mechanisms involved in the gene regulation, especially in light of recent experimental observations showing that mechanical forces applied to the nucleus can be sensed and processed by living cells, which can interpret the received mechanical signal, activating a number of specific genes in response to it [44,45,46,47].

In summary, the flexibility and advantages of the transfer-matrix formalism discussed above make it a powerful tool for a broad range of future applications, including, but not limited, to investigation of sequence-dependent DNA mechanical response, the study of local DNA structural transitions and the exploration of interactions between DNA and architectural proteins.

## Figures and Tables

**Table 1:** Structural and elastic characteristics of various DNA forms.

DNA form, *u*	Bending persistence	Twisting persistence	Contour length relative	DNA helical	Base-pairing energy relative	$\lambda_{u}$
	length, $L_{p}^{\left(u\right)}$ (nm)	length, $L_{\mathrm{tw}}^{\left(u\right)}$ (nm)	to B-DNA form	repeat, $h_{u}$ (bp)	to B-DNA form, $\mu_{u}$ ($k_{B}T$)	
B-DNA	50 [54,56]	95 [4,23,28]	1	10.4 [2]	0	4.3 [69]
L-DNA	7 [5,12]	15 [5,6,12]	1.35 [5,12]	16 [4,5,6]	5.0 [69]	4.3 [69]
P-DNA	15 [12]	25 [12]	1.7 [9,10,12]	3 [4,9,10,11,12]	17.8 [69]	−0.5 [69]
S-DNA	15 [12,83]	20 [12]	1.7 [4,8,9]	35 [4,9]	5.1 (this study)	4.3 (this study)

## Abbreviations

The following abbreviations are used in this manuscript:

DNA	deoxyribonucleic acid
bp	base-pair
FJC model	freely-jointed chain model
WLC model	worm-like chain model
sc-B, sc-L and sc-P	supercoiled B-, L- and P-DNA, respectively
B-to-L, B-to-P, B-to-S and L-to-S	structural transitions between the corresponding DNA states (B, L, P and S)
